# Rural-urban prescribing patterns by primary care and behavioral health providers in older adults with serious mental illness

**DOI:** 10.1186/s12913-022-08813-6

**Published:** 2022-11-29

**Authors:** Ulrike Muench, Matthew Jura, Cindy Parks Thomas, Jennifer Perloff, Joanne Spetz

**Affiliations:** 1 UCSF Department of Social and Behavioral Sciences, School of Nursing, University of California, Box 0612, 490 Illinois St., Floor 12, San Francisco, CA 94143 USA; 2 Philip R. Lee Institute for Health Policy Studies, School of Medicine, University of California, San Francisco, USA; 3 Healthforce Center, University of California, San Francisco, USA; 4 The Heller School, Brandeis University, Waltham, MA USA

**Keywords:** Serious mental illness, Advanced practice registered nurses, Psychiatric mental health nurse practitioners, Nurse practitioners, Primary care, Medicare

## Abstract

**Background:**

Older adults with serious mental illness (SMI) often have multiple comorbidities and complex medication schedules. Shortages of behavioral health specialists (BHSs), especially in rural areas, frequently make primary care providers (PCPs) the only clinician managing this complex population. The aim of this study was to describe rural/urban psychiatric medication prescribing in older adults with SMI by PCPs and BHSs, and by clinician type.

**Methods:**

This retrospective descriptive analysis used 2018 Medicare data to identify individuals with a bipolar, major depression, schizophrenia, or psychosis diagnosis and examined medication claims for antianxiety, antidepressants, antipsychotics, hypnotics, and anticonvulsants. Descriptive statistics summarized percentage of medications provided by PCPs and BHSs stratified by rural and urban areas and by drug class. Additional analyses compared psychiatric prescribing patterns by physicians, advanced practice registered nurses (APRNs), and physician assistants (PAs).

**Results:**

In urban areas, PCPs prescribed at least 50% of each psychiatric medication class, except antipsychotics, which was 45.2%. BHSs prescribed 40.7% of antipsychotics and less than 25% of all other classes. In rural areas, percentages of psychiatric medications from PCPs were over 70% for each medication class, except antipsychotics, which was 60.1%. Primary care physicians provided most psychiatric medications, between 36%-57% in urban areas and 47%-65% in rural areas. Primary care APRNs provided up to 13% of prescriptions in rural areas, which was more than the amount prescribed by BHS physicians, expect for antipsychotics. Psychiatric mental health APRNs provided up to 7.5% of antipsychotics in rural areas, but their prescribing contribution among other classes ranged between 1.1%-3.6%. PAs provided 2.5%-3.4% of medications in urban areas and this increased to 3.9%-5.1% in rural areas.

**Conclusions:**

Results highlight the extensive roles of PCPs, including APRNs, in managing psychiatric medications for older adults with SMI.

**Supplementary Information:**

The online version contains supplementary material available at 10.1186/s12913-022-08813-6.

## Introduction

As the population of older adults continues to grow and the Medicare-enrolled population is projected to grow from 54 million today to 77.5 million by 2030, so will the number of older adults with mental illness [1]. The prevalence of mental illness is estimated at 21% of all adults and serious mental illness (SMI), which includes bipolar disease, major depression and schizophrenia, is estimated to affect 14.2 million individuals or 5.6% of the U.S. population [2]. For individuals over 50 years of age, SMI reached its highest prevalence in 2020 at 3.4% [2], and evidence suggests that mental illness often goes undiagnosed in older adults [3–5].

Providing high quality care for older adults with behavioral health conditions is challenging for many reasons. Older adults often have multiple chronic conditions, and individuals with SMI have particularly high rates of comorbidities [6, 7] and complex medication schedules [8, 9]. Numerous studies have identified higher rates of cardiovascular disease, obesity, and metabolic syndrome in people with mental illness than in the general population. Alcohol and tobacco use, a lack of physical activity and poor sleep are additional behavioral risk factors contributing to a reduced life expectancy in people with mental illness [10, 11].

In the U.S., Medicare provides health insurance for individuals 65 years and older, including coverage for mental health needs. Preventative primary care and mental health services are available through Medicare Part B, such as screening for mental health disorders, individual and group psychotherapy, and medication management services [12]. Medicare Part A covers inpatient psychiatric services for up to 190 days. Prescription medications to treat mental health conditions are available under Medicare Part D. Part D is a voluntary, opt in, outpatient prescription drug benefit selected by approximately 48 million people of the more than 62 million fee-for-service Medicare enrollees who receive coverage through Medicare Parts A and B [12]. Medicare Part D consists of several plan options. Medications used to treat mental health conditions, such as antidepressants, anticonvulsants, and antipsychotics are generally available under all Part D plans. Alternatively, Medicare Advantage plans can be purchased, which offer additional services, such as counseling [12].

Even though research has consistently shown that individuals living with SMI experience co-existing chronic conditions that contribute to premature mortality [10], widespread shortages of behavioral health specialists (BHSs), including psychiatrists and psychiatric mental health nurse practitioners (PMHNPs) have created a lack of mental health services nationwide [13–16]. Prior research suggests that roughly two-thirds of primary care physicians are unable to connect their patients to outpatient behavioral health services [17]. Other research has found that Medicare beneficiaries with mental health conditions were less likely to receive comprehensive medication reviews, a medication management benefit offered under Medicare Part B [18]. The lack of BHSs, especially in rural areas [14, 19], means that primary care providers (PCPs) are often the primary source to access behavioral health care services [13, 20]. This includes the management of individuals with serious mental illness (SMI) and the prescribing of psychiatric medications.

To date, little is known about prescribing patterns by PCPs and BHSs for individuals with SMI. Understanding the contributions and practice patterns of these clinicians is important to ensuring adequate population mental health [21]. The objective of this study was to examine psychiatric medication prescribing in Medicare beneficiaries with SMI by PCPs and BHSs, by provider type, and by rural versus urban residence.

## Methods

We conducted a retrospective descriptive analysis of the entire fee-for-service Medicare population 65 years and older with continuous coverage in Parts A, B and D who had at least one prescription claim in 2018 and one inpatient or two outpatient claims of a primary bipolar, major depression, schizophrenia, or psychosis diagnosis. We selected continuous coverage to ensure that individuals had access to care and medication during our study period. While no standard diagnoses definition for SMI exist in the literature, we included bipolar, major depression, schizophrenia, or psychosis following prior research [22]. For identifying individuals living with SMI, we used the Center for Medicare & Medicaid Services (CMS) approach to identifying chronic conditions and selected individuals with one inpatient or two outpatient primary diagnoses claims in the respective diagnoses [23].

To identify prescriptions from primary care providers, behavioral health specialists, and providers working in other specialties we used the taxonomy code associated with the National Provider Identification (NPI) number on the prescription claim. This information is available in the prescriber characteristics file. We also used taxonomy codes to identify the type of provider (physician, advanced practice registered nurse (APRN), and physician assistant (PA)). Internal medicine, family medicine, general practice, and primary care were considered PCPs. Mental health, psychiatry, addiction medicine, and geropsychiatry were considered BHSs. Psychiatric mental health nurse practitioners were included in BHS, while adult, family, primary care, and geriatric APRN taxonomy codes were considered primary care. Physician assistants do not have formal specialties and were included in PCPs. Rural/urban location of beneficiaries was determined using zip code information on the master beneficiary summary file (MBSF) and applying Rural-Urban Commuting Area Codes (RUCA) [24].

Using the National Drug Code (NDC) on the prescription claims, we calculated the percentages of prescriptions from PCPs, BHSs, and other providers for medications commonly used in the treatment of SMI: [25] antianxiety, antidepressants, antipsychotics, hypnotics, and anticonvulsants, stratified by rural/urban setting. For a list of psychiatric medications belonging to these five drug classes that were prescribed for individuals in our sample, see Appendix Table 1. Prescriptions were standardized to 30 days supply. In additional analyses we identified the type of clinician to examine how prescribing patterns changed across specialties and provider type in urban and rural settings. Clinician types included physician, advanced practice registered nurse (APRN), physician assistant (PA), and other. Other providers include clinicians such as psychologists, who are allowed to prescribe in some states, and dentists.

To test whether the percentages of prescriptions differed in rural/urban areas we used a two-proportion z-test. Statistical significance was determined at conventional levels with alpha at 0.05. All analyses were conducted in SAS between November 2020 and January 2022. The University of California, San Francisco Institutional Review Board approved the study.

## Results

**Fig. 1:**
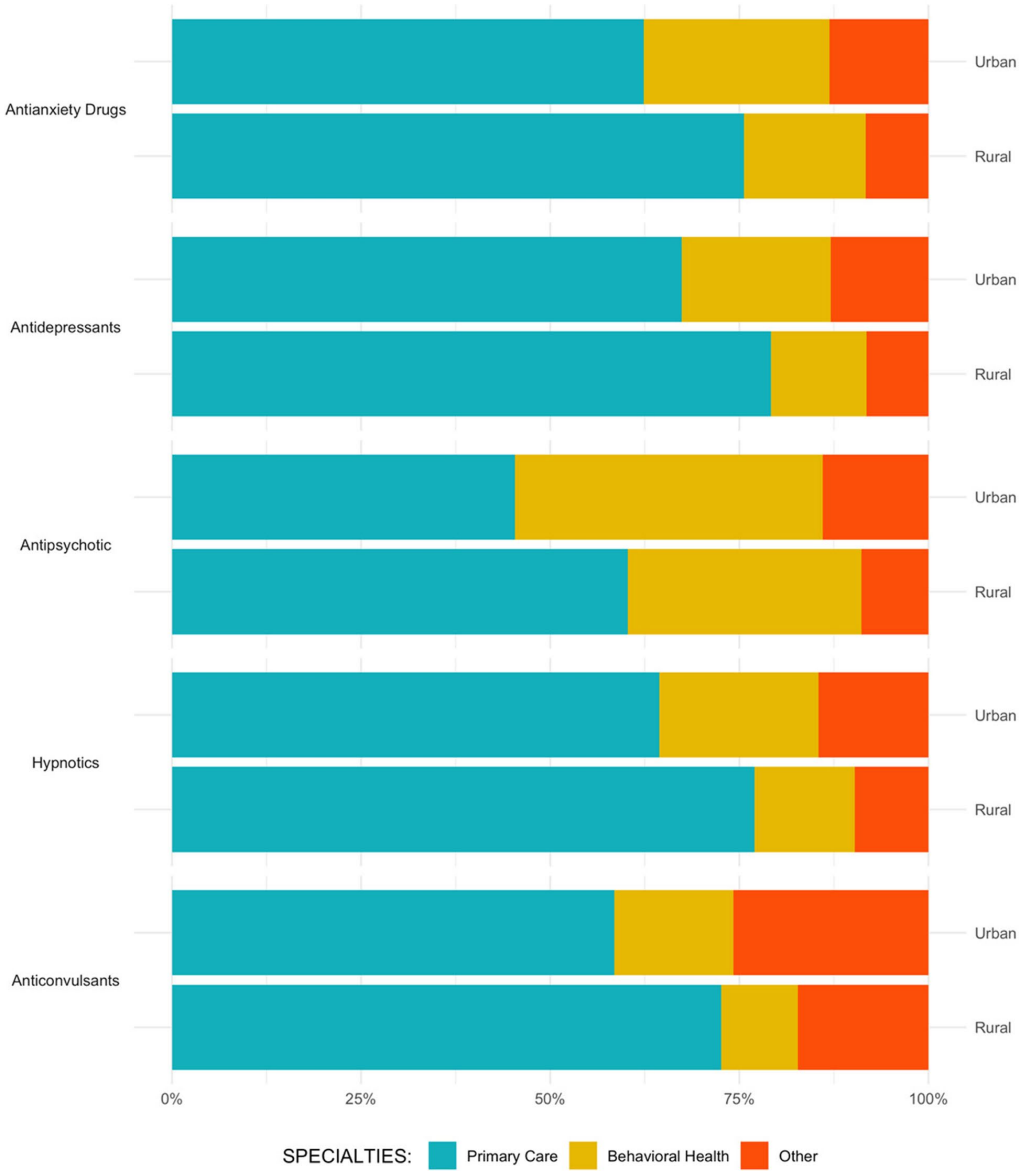
Rural/urban prescription percentages of psychiatric medications by provider specialty. Note: All urban-rural specialty differences are statistically significant at *p* <0.05

In 2018, there were 2,079,045 Medicare beneficiaries with a SMI diagnosis who received, on average, 24.2 30-day prescriptions from the identified drug classes. PCPs prescribed 64.0% of those medications, BHSs prescribed 19.1%, and other providers prescribed 15.9%. Figure 1 shows percentages of prescriptions from PCPs, BHSs, and other providers by drug class for rural and urban areas.

In urban areas, PCPs prescribed at least 50% of psychiatric medications for all classes except antipsychotics, with the highest percentages for antidepressants [67.4%; 95% CI, 67.35%-67.4%] and hypnotics [64.4%; 95% CI, 64.32%-64.5%]. BHSs prescribed 40.7% of antipsychotics [95% CI, 40.64%-40.75%] and less than 25% of all other classes. In rural areas, PCPs prescribed at least 60% of all psychiatric medication classes, with the highest percentages for antidepressants (79.2%; 95% CI, 79.15%-79.24%) and hypnotics (77%; 95% CI, 76.82%-77.24%). The medication classes for which BHSs had the greatest percentages in rural areas were antipsychotics (30.9%; 95% CI, 30.78%-31.03%) and hypnotics (13.2%; 95% CI, 13.05%-13.39%).

Table 1 presents medication percentages by type of PCP and BHS clinician for urban and rural areas. Physicians in primary care provided the largest percentages of psychiatric medications, ranging between 36%-57% per medication class in urban areas and 47%-64.7% in rural areas. Primary care APRNs in urban areas prescribed 6.1%-7.6% per drug class and in rural areas prescribed 8.9%-13.4%. PAs provided 2.5%-3.4% of medications in urban areas and this increased to 3.9%-5.1% in rural areas. BHS physician prescribing in urban areas was highest for antipsychotics and antianxiety medications with 34.0% and 20.9% respectively; they prescribed less than 20% for all other classes. In rural areas, percentages from BHS physicians for antipsychotics and antianxiety medications were substantially lower (22.8% and 12.2% respectively); other classes were 10% or lower. BHS APRNs prescribed in urban areas 5.9% of antipsychotics but only 0.9%-3.1% of other classes. Their percentages were similar in rural areas (1%-3.6%).

**Table 1:** Rural-urban prescription percentages of behavioral health drug classes and other medications by primary care specialty and provider type, with 95% CI

**Urban Areas**	**PCP MD**	**PCP APRN**	**PCP PA**	**PCP Other**	**BHS MD**	**BHS APRN**	**BHS Other**
**Behavioral Medications**							
Anti-anxiety drugs	51.42 (51.37-51.48)	7.58 (7.55-7.6)	3.25 (3.23-3.27)	0.13 (0.13-0.14)	20.92 (20.9-20.9)	3.11 (3.1-3.13)	0.49 (0.48-0.49)
Anti-depressants	57.09 (57.07-57.11)	6.84 (6.83-6.85)	3.32 (3.31-3.32)	0.12 (0.12-0.13)	16.8 (16.7-16.8)	2.5 (2.5-2.51)	0.43 (0.43-0.43)
Anti-psychotics	36.41 (36.36-36.46)	6.11 (6.09-6.14)	2.67 (2.65-2.69)	0.16 (0.16-0.16)	33.99 (33.9-34.1)	5.88 (5.85-5.91)	0.82 (0.81-0.83)
Hypnotics	55.22 (55.13-55.31)	6.02 (5.98-6.06)	3.08 (3.05-3.11)	0.09 (0.09-0.1)	18.7 (18.6-18.7)	2.03 (2.01-2.06)	0.31 (0.3-0.32)
Anti-convulsants	47.77 (47.74-47.81)	7.2 (7.19-7.22)	3.39 (3.38-3.41)	0.12 (0.12-0.13)	13.3 (13.2-13.4)	2.09 (2.08-2.1)	0.34 (0.34-0.34)
**Other Medications**	58.98 (58.97-58.99)	7.18 (7.18-7.19)	3.64 (3.63-3.64)	0.09 (0.09-0.09)	0.28 (0.28-0.28)	0.03 (0.03-0.03)	0.004 (0.004-0.004)
**Rural Areas**	**PCP MD**	**PCP APRN**	**PCP PA**	**PCP Other**	**BHS MD**	**BHS APRN**	**BHS Other**
**Behavioral Medications**							
Anti-anxiety drugs	57.16 (57.04-57.29)	13.37 (13.28-13.46)	4.89 (4.84-4.95)	0.2 (0.19-0.22)	12.16 (12.1-12.2)	3.59 (3.54-3.63)	0.3 (0.29-0.31)
Anti-depressants	61.24 (61.18-61.29)	12.65 (12.61-12.69)	5.14 (5.12-5.17)	0.16 (0.16-0.17)	9.45 (9.4-9.5)	2.89 (2.88-2.91)	0.26 (0.26-0.27)
Anti-psychotics	47.04 (46.91-47.18)	9.12 (9.04-9.19)	3.95 (3.9-4)	0.15 (0.14-0.16)	22.76 (22.6-22.9)	7.53 (7.46-7.6)	0.62 (0.6-0.64)
Hypnotics	59.2 (58.95-59.44)	12.71 (12.55-12.88)	4.92 (4.81-5.03)	0.2 (0.18-0.22)	10.16 (10.1-10.3)	2.73 (2.65-2.81)	0.33 (0.3-0.36)
Anti-convulsants	56.19 (56.1-56.27)	11.77 (11.72-11.83)	4.47 (4.43-4.51)	0.17 (0.16-0.18)	7.64 (7.6-7.7)	2.27 (2.24-2.29)	0.22 (0.21-0.23)
**Other Medications**	62.14 (62.12-62.17)	12.99 (12.97-13)	5.18 (5.17-5.19)	0.14 (0.14-0.14)	0.21 (0.21-0.21)	0.05 (0.05-0.05)	0.01 (0.01-0.01)

1) Abbreviations: PCP MD: Primary care provider physician; PCP APRN: Primary care provider advanced practice registered nurses; Primary care provider physician assistant; Behavioral health specialist physician, Behavioral health specialist advanced practice registered nurse

2) Row percent do not add up to 100 because other clinician specialties are not shown

3) Specialty and provider type were identified using taxonomy codes from the prescriber characteristics file

4) All urban/rural differences significant at p <0.001

5) PAs do not subspecialize and are identified within PCP only

6) Other PCP providers included certified nurse midwifes, certified clinical nurse specialists. Other BHS included certified nurse specialists in mental health. Other providers (not shown) included dentists, psychologist, certified nurse anesthetists. Other specialties included for example, sports medicine, emergency medicine, gynecology etc

## Discussion

Across major drug classes used to treat SMI, most psychiatric medications were prescribed by PCPs. In rural areas, PCPs prescribed over 75% of the medications in four of the six drug classes examined, including hypnotics and psychotherapeutics, which are specialized psychiatric medications. Providers with behavioral health expertise provided more psychiatric medications in urban areas, at 40% for antipsychotics but less than 25% for other psychiatric medications. In rural areas, percentages from BHSs were significantly smaller, at 30% for antipsychotics and under 16% for other psychiatric medications. The remaining medications were provided by providers with other specialties, including physicians in general surgery, neurology, rheumatology, and psychologists and dentists.

The American Academy of Family Physicians (AAFP) states in their position paper on mental health care services that “family physicians are well-prepared to provide many mental health services” [26]. Our findings demonstrate that primary care physicians are indeed playing a large role in this area of care, prescribing more than half of all psychiatric medications. Primary care APRNs were also substantially involved in prescribing psychiatric medications to Medicare beneficiaries with SMI, especially in rural areas, where they provided up to 13% of medications, including twice as many medications for antianxiety, antidepressants and hypnotics compared to urban areas. In rural areas, primary care APRNs prescribed 2-4 times as many medications across psychiatric drug classes than behavioral health specialty physicians.

The extensive involvement of primary care physicians and APRNs in managing psychiatric medications for patients with SMI in rural areas may, in part, reflect a lack of access to behavioral health specialists. Prior research found that primary care providers who practiced in counties with fewer psychiatrists were significantly more likely to report that they could not find outpatient mental health services for their patients [17]. Our analysis did not identify if the PCPs sought to obtain specialist behavioral health services and cannot determine the extent to which the prescribing patterns observed by PCPs in some areas may have been driven by poor access to behavioral health specialists.

With large projected increases in the numbers of older adults with SMI in the coming years, the roles of primary care providers in the delivery of care for this population are likely to grow. Previous research has indicated that PCPs often feel underprepared to provide services for patients with complex behavioral health needs [27, 28]. Difficult medication schedules [9], multiple comorbidities, longer visit times, frequent follow-up care [29], and communication challenges [27] can make providing care for individuals with SMI especially demanding. For this reason, payment mechanisms that adequately reimburse primary care physicians and APRNs for the care of people with SMI should be instituted.

Our findings revealed that the role of psychiatric mental health APRNs in the prescribing of medications for older adults with SMI was limited compared to their colleagues in primary care. This provides a workforce opportunity to invest in the growth of psychiatric mental health nurse practitioners (PMHNP) who are trained to provide care to individuals with a wide variety of mental illnesses [30]. A recent study examining billing trends in Medicare Part D of psychiatrist and PMHNPs found that between 2013 and 2019 the number of counties with only a psychiatrist billing for Part D services decreased from 277 to 178 and the number of counties with only a PMHNPs increased from 81 to 156. Yet, 857 rural counties had neither a psychiatrist nor PMHNPs prescribing medications [31]. While the growth in numbers of PMHNPs prescribing medications in rural areas is promising, our results suggest that in comparison to primary care APRNs, PMHNPs provide a small proportion of medications to Medicare beneficiaries with SMI.

To optimally utilize the psychiatric mental health nurse practitioner workforce towards meeting population mental health needs, studies are needed that guide primary care practices in how best to integrate these specialized clinicians [21]. Furthermore, research is needed that evaluates quality measures of behavioral health prescribing and other primary care outcomes with different clinician compositions as part of the primary care team. State scope of practice regulations for APRNs that require physician oversight for practice and prescribing even for experienced APRNs may be a barrier to accessing mental health care. Alexander and Schnell found that when APRNs can prescribe independently, counties that are underserved for mental health care resources have greater use of antidepressants and antipsychotics relative to when APRNs must have physician oversight [32]. They also found better patient-reported mental health outcomes in states where APRNs can prescribe independently.

Our study had a number of limitations, including potentially outdated taxonomy information as providers may delay updating their board certification, potential misattribution of prescriptions due to Medicare rules that allow some APRN services to be billed by a physician [33], and that our analysis was descriptive and, thus, did not control for beneficiary, or any other characteristics. Further, our analysis identified SMI as diagnoses from bipolar disorder, major depression, schizophrenia, or psychosis. Other definitions of SMI could have resulted in different results. Finally, this analysis is based on Medicare claims data and findings might not be generalizable to other populations.

In summary, our results highlight the extensive roles of PCPs, including APRNs, in managing medications for older Medicare beneficiaries with SMI. Providing educational opportunities that prepare for the management of these complex patients and clinical support mechanisms seems warranted. Furthermore, as the demand for primary care clinicians with behavioral health expertise is continuing to grow, strategies aimed at growing the psychiatric mental health nurse practitioner workforce could be one avenue to increase access to BHSs, particularly in rural areas where APRNs are essential to accessing health care services.

## Supplementary Information

**Additional file 1.**

## Data Availability

The data that support the findings of this study are available from CMS [34] (resdac@umn.edu) but restrictions apply to the availability of these data, which were used under license for the current study, and so are not publicly available.

## Abbreviations

SMI: Serious mental illness
BHS: Behavioral health specialists
PCP: Primary care providers
MD: Physician
APRN: Advanced practice registered nurses
PA: Physician assistant
