# Supplementary material for: Rural-urban prescribing patterns by primary care and behavioral health providers in older adults with serious mental illness
Source: BMC Health Serv Res. 2022 Nov 29;22:1440. doi: 10.1186/s12913-022-08813-6 (PMC9706942; doi:10.1186/s12913-022-08813-6)
Supplement: Supplementary file 1 — Additional file 1. [file 12913_2022_8813_MOESM1_ESM.docx]

Appendix: Rural-urban prescribing patterns by primary care and behavioral health providers in older adults with serious mental illness.

Table 1: List of medications taken by individuals with SMI as defined by our selection criteria, by drug class

| Antianxiety | |
| --- | --- |
|  | Alprazolam |
|  | Buspirone HCl |
|  | Chlordiazepoxide HCl |
|  | Clorazepate Dipotassium |
|  | Diazepam |
|  | Hydroxyzine HCl |
|  | Hydroxyzine Pamoate |
|  | Lorazepam |
|  | Meprobamate |
|  | Oxazepam |
|  |  |
| Antidepressants | |
|  | Amitriptyline HCl |
|  | Amoxapine |
|  | Bupropion HCl |
|  | Bupropion Hydrobromide |
|  | Citalopram Hydrobromide |
|  | Clomipramine HCl |
|  | Desipramine HCl |
|  | Desvenlafaxine |
|  | Doxepin HCl |
|  | Duloxetine HCl |
|  | Escitalopram Oxalate |
|  | Fluoxetine HCl |
|  | Fluvoxamine Maleate |
|  | Imipramine HCl |
|  | Imipramine Pamoate |
|  | Isocarboxazid |
|  | Levomilnacipran HCl |
|  | Maprotiline HCl |
|  | Mirtazapine |
|  | Nefazodone HCl |
|  | Nortriptyline HCl |
|  | Paroxetine HCl |
|  | Paroxetine Mesylate |
|  | Phenelzine Sulfate |
|  | Protriptyline HCl |
|  | Selegiline |
|  | Sertraline HCl |
|  | Tranylcypromine Sulfate |
|  | Trazodone HCl |
|  | Trimipramine Maleate |
|  | Venlafaxine HCl |
|  | Vilazodone HCl |
|  | Vortioxetine HBr |
|  |  |
| Antipsychotics | |
|  | Aripiprazole |
|  | Aripiprazole Lauroxil |
|  | Asenapine Maleate |
|  | Brexpiprazole |
|  | Carbamazepine (Antipsychotic) |
|  | Cariprazine HCl |
|  | Chlorpromazine HCl |
|  | Clozapine |
|  | Fluphenazine Decanoate |
|  | Fluphenazine HCl |
|  | Haloperidol |
|  | Haloperidol Decanoate |
|  | Haloperidol Lactate |
|  | Iloperidone |
|  | Lithium |
|  | Lithium Carbonate |
|  | Lithium Citrate |
|  | Loxapine Succinate |
|  | Lurasidone HCl |
|  | Molindone HCl |
|  | Olanzapine |
|  | Olanzapine Pamoate |
|  | Paliperidone |
|  | Paliperidone Palmitate |
|  | Perphenazine |
|  | Pimavanserin Tartrate |
|  | Prochlorperazine |
|  | Prochlorperazine Edisylate |
|  | Prochlorperazine Maleate |
|  | Quetiapine Fumarate |
|  | Risperidone |
|  | Risperidone Microspheres |
|  | Thioridazine HCl |
|  | Thiothixene |
|  | Trifluoperazine HCl |
|  | Ziprasidone HCl |
|  | Ziprasidone Mesylate |
|  |  |
| Hypnotics |  |
|  | Butabarbital Sodium |
|  | Doxepin HCl |
|  | Estazolam |
|  | Eszopiclone |
|  | Flurazepam HCl |
|  | Midazolam HCl |
|  | Phenobarbital |
|  | Ramelteon |
|  | Secobarbital Sodium |
|  | Suvorexant |
|  | Tasimelteon |
|  | Temazepam |
|  | Triazolam |
|  | Zaleplon |
|  | Zolpidem Tartrate |
|  |  |
| Anticonvulsants | |
|  | Brivaracetam |
|  | Carbamazepine |
|  | Clobazam |
|  | Clonazepam |
|  | Diazepam |
|  | Divalproex Sodium |
|  | Eslicarbazepine Acetate |
|  | Ethosuximide |
|  | Ethotoin |
|  | Ezogabine |
|  | Felbamate |
|  | Fosphenytoin Sodium |
|  | Gabapentin |
|  | Lacosamide |
|  | Lamotrigine |
|  | Levetiracetam |
|  | Methsuximide |
|  | Oxcarbazepine |
|  | Perampanel |
|  | Phenytoin |
|  | Phenytoin Sodium |
|  | Phenytoin Sodium Extended |
|  | Pregabalin |
|  | Primidone |
|  | Rufinamide |
|  | Tiagabine HCl |
|  | Topiramate |
|  | Valproate Sodium |
|  | Valproic Acid |
|  | Zonisamide |
